# Supplementary material for: Wealth-based inequalities in early childhood development (ECD) outcomes in Bangladesh: A decomposition analysis using MICS 2019
Source: PLOS Glob Public Health. 2025 Jul 2;5(7):e0004774. doi: 10.1371/journal.pgph.0004774 (PMC12221076; doi:10.1371/journal.pgph.0004774)
Supplement: S1 Text — (DOCX) [file pgph.0004774.s001.docx]

**Supporting information**

**Supplementary Table 1.** List of variables identified from literature and can be calculated using Bangladesh Multiple Indicator Cluster Survey (MICS) 2019 dataset.

| **SN** | **Variable** | **Measurement** | **References** |
| --- | --- | --- | --- |
| 1 | Age (in months) | Categorized as: 36-47 (0); 48-59 (1) | [1–3] |
| 2 | Sex of child | Categorized as: Male (0); Female (1) | [1–3] |
| 3 | Nutritional Status | There are several weight and height measurements available in MICS 2019 to categorize the nutritional status of children. We have used weight for age Z score (WAZ) as a proxy for the nutritional status and categorized as:  Severely malnourished: WAZ < -3.0 (0);  Moderately malnourished: -3.0 < WAZ < -2.01 (1);  Nourished: WAZ > -2.01 (2) | [2, 4] |
| 4 | Attendance to early childhood education | Categorized as: No (0); Yes (1) | [2, 3] |
| 5 | Early childhood diseases | Categorized as: No (0); Yes (1) | [4–6] |
| 6 | Child has book at home | Categorized as: No (0); Yes (1) | [2, 3] |
| 7 | Type of residence | Categorized as: Urban (0); Rural (1) | [2, 3, 7] |
| 8 | Division | Categorized as: Barisal (0); Chittagong (1); Dhaka (2); Khulna (3); Mymensingh (4); Rajshahi (5); Rangpur (6); Sylhet (7) | [1, 5, 8] |
| 9 | Mother’s education | Categorized as: Pre-primary or none (0); Primary (1); Secondary (2); Higher secondary+ (3) | [1, 3, 8] |
| 10 | Childcare involvement of mother | Categorized as: No (0); Yes (1) | [4, 6] |
| 11 | Improper supervision | Categorized as: No (0); Yes (1) | [1, 5, 6] |
| 12 | Wealth index quintile (for decomposition analysis) | Categorized as: poorest (0); second (1); middle (2); fourth (3); richest (4) | [1–3] |

**Supplementary Table 2.** Variance Inflation Factors (VIFs) to check multicollinearity of the variables in the model.

| **Variables** | **df** | **VIF** | **1/VIF** |
| --- | --- | --- | --- |
| Age (in months) | 1 | 1.452 | 0.689 |
| Sex of child | 1 | 1.37 | 0.73 |
| Nutritional Status | 2 | 1.157 | 0.864 |
| Attendance to early childhood education | 1 | 1.157 | 0.864 |
| Early childhood diseases | 1 | 1.156 | 0.865 |
| Child has book at home | 1 | 1.153 | 0.867 |
| Type of residence | 1 | 1.062 | 0.941 |
| Division | 7 | 1.037 | 0.964 |
| Mother’s education | 3 | 1.024 | 0.977 |
| Multiple Childcare Involvement of mother | 1 | 1.015 | 0.985 |
| Improper supervision | 1 | 1.008 | 0.992 |
| Wealth index quintile | 4 | 1.005 | 0.995 |
| **Mean VIF** |  | **1.133** |  |

**References**

1. Alam MI, Mansur M, Barman P. Early childhood development in Bangladesh and its socio-demographic determinants of importance. Early Child Dev Care. 2022;192:1901–20.

2. Hossain MI, Haq I, Zinnia MA, Mila MS, Nayan MIH. Regional variations of child development index in Bangladesh. Heliyon. 2021;7.

3. Islam MM, Khan JR, Kabir A, Khan MZR, Islam MM. Associations of Socio-Demographic and Environmental Factors with the Early Development of Young Children in Bangladesh. International Journal of Early Childhood. 2021;53:175–96.

4. Walker SP, Wachs TD, Grantham-Mcgregor S, Black MM, Nelson CA, Huffman SL, et al. Inequality in early childhood: Risk and protective factors for early child development. The Lancet. 2011;378:1325–38.

5. Hasan MN, Babu MdR, Chowdhury MAB, Rahman MM, Hasan N, Kabir R, et al. Early childhood developmental status and its associated factors in Bangladesh: a comparison of two consecutive nationally representative surveys. BMC Public Health. 2023;23:687.

6. D P. Factors Affecting Early Childhood Growth and Development: Golden 1000 Days. Advanced Practices in Nursing. 2016;01.

7. Haq I, Hossain MI, Zinnia MA, Hasan MR, Chowdhury IAQ. Determinants of the Early Childhood Development Index among children aged < 5 years in Bangladesh, Costa Rica and Ghana: a comparative study. Eastern Mediterranean Health Journal. 2021;27:1069–77.

8. Rahman F, Tuli SN, Mondal P, Sultana S, Hossain A, Kundu S, et al. Home environment factors associated with early childhood development in rural areas of Bangladesh: evidence from a national survey. Front Public Health. 2023;11.
