# Supplementary material for: Wealth-based inequalities in early childhood development (ECD) outcomes in Bangladesh: A decomposition analysis using MICS 2019
Source: PLOS Glob Public Health. 2025 Jul 2;5(7):e0004774. doi: 10.1371/journal.pgph.0004774 (PMC12221076; doi:10.1371/journal.pgph.0004774)
Supplement: Attachment — Submitted filename: review report_PGPH-D-25-00061.pdf [file pgph.0004774.s003.pdf]

**Wealth-based Inequalities in Early Childhood Development (ECD) Outcomes in Bangladesh: A Decomposition Analysis Using MICS 2019**

Review Report

This manuscript investigates wealth-based inequalities in Early Childhood Development (ECD) outcomes in Bangladesh using data from the 2019 Multiple Indicator Cluster Survey (MICS). The study employs concentration indices, concentration curves, and decomposition analysis to quantify and understand disparities between poor and non-poor groups. The manuscript is well-structured. However, I would like to raise the following queries for the authors.

1. Page 8, line 208: $d_{ir}$ is not defined.

2. Page 9, line 263: the variable is 'binary' other than 'binomial'.

3. Page 10 and 11: The notation in equations (1), (2), and (3) is not defined. For instance, it is unclear what $p$, $r$ and $h$ represent.

4. Some $p$-values are reported with excessive decimal places. They should be rounded appropriately.

5. The significance of some findings, particularly for social-emotional and physical domains, should be discussed more critically since the concentration indices are relatively small.

6. Wealth-based disparities in ECD have been identified in previous studies (e.g., reference [24] in the paper). What new insights does this study contribute beyond existing findings?
