# Supplementary material for: Wealth-based inequalities in early childhood development (ECD) outcomes in Bangladesh: A decomposition analysis using MICS 2019
Source: PLOS Glob Public Health. 2025 Jul 2;5(7):e0004774. doi: 10.1371/journal.pgph.0004774 (PMC12221076; doi:10.1371/journal.pgph.0004774)
Supplement: Attachment — Submitted filename: Response to Reviewers.docx [file pgph.0004774.s004.docx]

Paper Title: **Wealth-Based Inequalities in Early Childhood Development (ECD) Outcomes in Bangladesh: A Decomposition Analysis Using MICS 2019**

Dear Editor,

We have tried our best to address the queries and incorporate the suggestions from you. We have prepared a table in which the left-side column includes your comments, and the right-side column includes the reply to the comments.

Thank you very much for your valuable suggestions. If any further clarification or improvement is required, please let us know — we would be happy to address your queries.

Regards,

Anonymous Authors

| Comments from Editor / Reviewers | Reply from the Authors |
| --- | --- |
| **Editor** | |
| The current study intends to assess the ECD inequality across wealth groups in Bangladesh. However, it is already known that wealth plays a critical role in ECD in Bangladesh. For example, Hasan et al. (2023) using the MICS data reported that “children from affluent families had 1.32- and 1.26 times higher odds- on track than those from the poorest families.” Similar findings were reported by Rahman et al. (2023), “compared to the poor counterpart, children from households with rich wealth status had better chance to attain overall ECD status, particularly in the literacy-numeracy and learning domains.” As such, it remained unclear that what the novel contribution of the current paper is to the literature. | Thank you for your careful reading of our manuscript and for your insightful and constructive comments. We truly appreciate the time and effort you have taken to provide valuable feedback.  While previous studies, such as Hasan et al. (2023) and Rahman et al. (2023) have established the association between wealth and ECD outcomes in Bangladesh, these analyses primarily focused on determining the factors affecting ECD and did not explore wealth-based inequalities in ECD across all four domains using concentration curves or indices. Moreover, we applied a standard decomposition approach to disentangle and quantify the contribution of individual socioeconomic factors to the observed disparities.  To specify the novel contributions of the current paper to the literature, we have modified the background section. A newly added paragraph is put below:  This paper contributes to the literature in three notable respects. First, it examines wealth-based inequality across all four domains of ECD—literacy-numeracy, physical, socio-emotional, and learning—using concentration indices and concentration curves. Second, it applies a standard decomposition method to quantify the contribution of specific socioeconomic and demographic factors to the observed inequalities. Third, by analyzing domain-specific disparities, the study offers targeted insights for designing policy interventions to improve ECD outcomes and reduce inequities in Bangladesh.  The changes in the background section are highlighted in turquoise color. |
| The current study estimated a concentration index and employed a decomposition method. It was stated that “a popular approach for determining the causes of inequality is the decomposition method developed by [52]”. However, the cited paper of Wagstaff and Doorslaer (2003, Health Econ) has nothing to do with decomposition. Wagstaff et al. had other papers which were about decomposition. For example, Wagstaff et al. (2003, JHE, https://doi.org/10.1016/S0304-4076(02)00161-6). Please carefully review the correct citation and perform the decomposition analysis correctly. | Thank you for pointing out this significant referencing error in the Methodology section.  We have replaced the citation (highlighted in yellow), and necessary editing has also been done. |
| Why the wealth index quintiles are part of decomposition, when the concentration index was based on wealth scores? | Even though the concentration index is based on wealth scores (a continuous variable), wealth quintiles (categorical groups) can also be used in decomposition for several reasons. Primarily, the goal of decomposition is to explain the inequality in a health outcome (e.g., ECD) with respect to wealth, not to explain the wealth ranking itself.  Including wealth index quintiles as explanatory variables allows us to assess how differences in health outcomes are associated with different socioeconomic groups and to quantify how much of the observed inequality is attributable to wealth.  While the concentration index uses continuous wealth scores to rank individuals, the decomposition treats wealth (in quintiles) as a covariate, helping determine its contribution to the inequality through its influence on the outcome and its unequal distribution across the population.  A few references where wealth index quintiles are included in the decomposition of the concentration index are mentioned below: 1.  [Decomposing inequality in Maternal and Child Health (MCH) services in Nepal \| BMC Public Health \| Full Text](https://bmcpublichealth.biomedcentral.com/articles/10.1186/s12889-023-15906-2)  2.  [Socioeconomic inequalities in utilizing facility delivery in Bangladesh: A decomposition analysis using nationwide 2017–2018 demographic and health survey data \| PLOS One](https://journals.plos.org/plosone/article?id=10.1371/journal.pone.0278093) |
| In general, make a careful case about why this study is contributing to the literature. Remove repetition of extant study findings. Explicitly communicate the meaning of the decomposition analysis and link those to potential policy implications. | A paragraph has been added at the end of the background section, mentioning the specific contributions of the current study to the literature.  The background, discussion (highlighted in yellow), and conclusion (highlighted in green) sections have also been modified according to your suggestions. Thank you very much once again. |
| **Reviewer #1** | |
| This study focuses on disparities in early childhood development among different socioeconomic groups in Bangladesh. It uses concentration curves, decomposition analysis, and logistic regression analysis to identify various factors associated with ECD. The methodology and results are clearly explained in the manuscript. However, I think it is not necessary to explain what the odds ratio is. | Thank you very much for reviewing our manuscript.  The explanation of the odds ratio has been shortened and modified (highlighted in yellow). |
| In the discussion section, the “limitations” of this study could be mentioned as it is a secondary analysis. Also, possible mechanisms related to wealth status and ECD could be better explained, as they are the most important findings of this study. | The limitations of the study have been included at the end of the discussion section (highlighted in yellow).  The discussion section has also been modified. |
| Overall, the manuscript is well structured and presents strong, well-researched, and methodologically sound research. However, the “Introduction” and “Conclusion” sections could be made more concise. | The background and conclusion sections have now been modified (highlighted in turquoise and green) according to your suggestions. Thank you very much once again. |
| **Reviewer #2** | |
| This manuscript investigates wealth-based inequalities in Early Childhood Development (ECD) outcomes in Bangladesh using data from the 2019 Multiple Indicator Cluster Survey (MICS). The study employs concentration indices, concentration curves, and decomposition analysis to quantify and understand disparities between poor and non-poor groups. The manuscript is well-structured. | We sincerely thank you for your thorough review of our manuscript and your insightful observations. |
| However, I would like to raise the following queries for the authors.   1. Page 8, line 208: *d_ir_* is not defined. | *d_ir_* is now defined and explained in the manuscript.  The changes are highlighted in bright green. |
| 2. Page 9, line 263: the variable is ‘binary' other than ‘binomial'. | The error has been rectified in the manuscript. |
| 3. Page 10 and 11: The notation in equations (1), (2), and (3) is not defined. For instance, it is unclear what *p*, *r,* and *h* represent. | The notations in equations (1), (2), and (3) have been defined and explained. The changes are highlighted in bright green. |
| 4. Some *p*-values are reported with excessive decimal places. They should be rounded appropriately. | All *p-*values are now rounded and reported up to 3 decimal places. |
| 5. The significance of some findings, particularly for social-emotional and physical domains, should be discussed more critically since the concentration indices are relatively small. | An explanation has been provided by adding a paragraph (highlighted in yellow) in the discussion section of the manuscript. |
| 6. Wealth-based disparities in ECD have been identified in previous studies (e.g., reference [24] in the paper). What new insights does this study contribute beyond existing findings? | The background section now concludes with a paragraph that mentions how this study contributes beyond existing findings (highlighted in turquoise).  While the existing studies primarily focused on determining the factors affecting ECD, our study contributes to the literature in three key ways. First, it examines wealth-based inequality across all four domains of ECD—literacy-numeracy, physical, socio-emotional, and learning—using concentration indices and concentration curves. Second, it applies a standard decomposition method to quantify the contribution of specific socioeconomic and demographic factors to the observed inequalities. Third, by analyzing domain-specific disparities, the study offers targeted insights for designing policy interventions to improve ECD outcomes and reduce inequities in Bangladesh.  Thank you once again for your sharp observations. It helped us a lot to revise and refine our manuscript. |

**********
