# Supplementary material for: Wealth-based inequalities in early childhood development (ECD) outcomes in Bangladesh: A decomposition analysis using MICS 2019
Source: PLOS Glob Public Health. 2025 Jul 2;5(7):e0004774. doi: 10.1371/journal.pgph.0004774 (PMC12221076; doi:10.1371/journal.pgph.0004774)
Supplement: Attachment — Submitted filename: Response_to_Reviewers_auresp_2.docx [file pgph.0004774.s005.docx]

Paper Title: **Wealth-Based Inequalities in Early Childhood Development (ECD) Outcomes in Bangladesh: A Decomposition Analysis Using MICS 2019**

Dear Editor,

We have tried our best to address the queries and incorporate the suggestions from you. We have prepared a table in which the left-side column includes your comments, and the right-side column includes the reply to the comments.

Thank you very much for your valuable suggestions. If any further clarification or improvement is required, please let us know — we would be happy to address your queries.

Regards,

Anonymous Authors

| Comments from Editor / Reviewers | Reply from the Authors |
| --- | --- |
| **Editor** | |
| Thank you for submitting a revised version of the manuscript. It appears that the key contribution of this manuscript is to demonstrate wealth-based inequalities and decompose the role of contributing factors to the “wealth-based” inequality. However, the fundamental error in the analysis is that “wealth index quintiles” were also considered as contributing factors to “wealth-based” inequalities. The key assumption of the seminal paper on decomposition of inequality by Wagstaff et al. (2003, Journal of Econometrics) was that “everyone in the selected sample or subsample—irrespective of their income—faces the same coefficient vector” for the determinants of the outcome variable and “interpersonal variations in the outcome are thus assumed to derive from systematic variations across income groups in the determinants.”  As such, inclusion of “wealth index quintiles” as determinants of the outcomes is seriously erroneous as there is no way that everyone in the sample, irrespective of their income, will have the same coefficients for wealth index quintiles. The argument stated by the authors that “how much of the observed inequality is attributable to wealth”, literally makes no sense in the context of the decomposition technique. The two references cited are subject to same mistake, and unfortunately were not carefully adjudicated by the reviewers and the editors.  Please revise the analysis, following the proper methods. Please do not get misled by erroneous publications like those mentioned in the response to the review comments.  The decomposition, however, can be done for sub-groups of wealth if the authors are interested to explore. See Clarke et al. (2003, Health Economics) for details. | Thank you very much for your insightful observation and for pointing out this critical issue in our analysis.  We sincerely appreciate your close review of our manuscript and your reference to the foundational work by Wagstaff et al. (2003) and other studies. Your observation regarding the inappropriate inclusion of “wealth index quintiles” as a determinant in the decomposition of wealth-based inequality is highly pertinent and well taken.  We have carefully considered your guidance and, as advised, have excluded the wealth index quintile from the set of explanatory variables in our decomposition analysis. The revised analysis has been conducted accordingly to ensure methodological consistency (highlighted in yellow).  Thank you again for your thoughtful contribution. It has significantly strengthened the analytical rigor of our study. We are grateful for your time and expertise in reviewing this manuscript. |

**********
